# Heroin pipe distribution to reduce high-risk drug consumption behaviors among people who use heroin: a pilot quasi-experimental study

**DOI:** 10.1186/s12954-022-00685-7

**Published:** 2022-09-22

**Authors:** Thomas Fitzpatrick, Vanessa M. McMahan, Noah D. Frank, Sara N. Glick, Lauren R. Violette, Shantel Davis, Shilo Jama

**Affiliations:** 1 Division of Allergy and Infectious Diseases, Department of Medicine, University of Washington, Seattle, WA USA; 2 The People’s Harm Reduction Alliance, 1959 NE Pacific St., Box 356423, Seattle, WA 98195 USA; 3 Center on Substance use and Health, Department of Public Health, San Francisco, CA USA; 4 Office of Infectious Disease, Washington State Department of Health, Olympia, WA USA; 5 Department of Epidemiology, University of Washington, Seattle, WA USA; 6 Safer Alternatives Thru Networking and Education, Sacramento, CA USA

**Keywords:** Heroin pipe, Route transition intervention, Heroin smoking, Heroin injection, People who use drugs

## Abstract

**Background:**

Heroin pipe distribution may encourage people who use heroin (PWUH) to transition from injecting to smoking heroin, reducing harms associated with injection drug use. A syringe services program (SSP) in Seattle, Washington, led by people who use drugs developed a heroin pipe distribution program.

**Methods:**

We conducted a pretest–posttest quasi-experimental study to evaluate the impact of heroin pipe distribution on drug consumption behaviors among PWUH between March and December 2019. SSP clients were surveyed during three weeklong timepoints before and four weeklong timepoints after heroin pipe distribution. Primary outcomes were change in proportion of SSP clients who exclusively injected heroin, exclusively smoked heroin, and both injected and smoked heroin in the past seven days comparing the pre- and post-intervention periods.

**Results:**

Across the seven observation timepoints, 694 unique respondents completed 957 surveys. Multiple responses from a single respondent in a given period were collapsed, resulting in 360 pre-intervention and 430 post-intervention records. Heroin use was reported in over half of pre-intervention (56%, 201/360) and post-intervention records (58%, 251/430). Compared to pre-intervention behaviors, the proportion of respondents who exclusively injected heroin was lower after the start of heroin pipe distribution (32%, 80/251 vs 43%, 86/201, *p* = 0.02), while the proportion of respondents who both injected and smoked heroin was higher (45%, 113/251 vs 36%, 72/201, *p* = 0.048). Just under half (44%, 110/251) of respondents who used heroin during the post-intervention period used a heroin pipe obtained from the SSP, of which 34% (37/110) reported heroin pipe distribution had reduced their heroin injection frequency. Self-reported hospitalization for a pulmonary cause was not associated with using a heroin pipe.

**Conclusions:**

The proportion of SSP clients who exclusively injected heroin was lower after implementation of heroin pipe distribution. Randomized studies with longer follow-up are needed to investigate whether heroin pipe distribution reduces heroin injection and improves health outcomes associated with drug use. Limited intervention exposure, loss to follow-up, and pipe availability from other sources pose methodological challenges to evaluations of route transition interventions in community settings. This pilot highlights the potential for organizations led by people who use drugs to develop, implement, and evaluate novel public health programming.

**Supplementary Information:**

The online version contains supplementary material available at 10.1186/s12954-022-00685-7.

## Background

People who use heroin (PWUH) have increased morbidity and mortality compared to the general population [1]. A syndemic of opioid overdose, human immunodeficiency virus (HIV), hepatitis C virus (HCV), skin and soft tissue infections (SSTI), and infective endocarditis accounts for many of the poor health outcomes among PWUH [2–5]. Heroin can be consumed in several ways, including injection and smoking [6]. High-risk injection behaviors, including syringe sharing and reuse of non-sterile injection equipment, are established routes of HIV and HCV transmission and increase risk of SSTI and infective endocarditis [7–9]. Opioid overdose is a common cause of mortality among PWUH, with higher overdose risk among those who inject [10–12].

Because smoking heroin does not injure the skin or introduce non-sterile equipment into blood or tissue, this method of consumption does not entail the same risk of blood-borne infections or SSTI compared to injection. While similar pharmacological effects can be achieved by smoking or injecting heroin, peak plasma concentrations are 2–4 times lower when heroin is smoked, which may reduce risk of lethal opioid overdose [13, 14]. Programs that encourage PWUH to transition from injecting to smoking heroin may decrease injection frequency and thereby reduce harms associated with heroin use, including risks of infection and overdose [15]. Distribution of smoking equipment may also help PWUH avoid using pipes fashioned from cans or other poor-quality materials that easily crack or overheat, thereby reducing risk of developing burns or cuts on the lips that can serve as sites of infection [16–18]. Pipe distribution programs may also reduce pipe sharing, a risk behavior potentially associated with respiratory virus or HCV transmission [17–20].

The potential for route transition interventions (RTIs) to improve health outcomes among people who use drugs (PWUD) was first identified in the 1990s [21]. Since then, programs that distribute methamphetamine and crack cocaine pipes have become increasingly common in Europe and North America [22]. Evaluations of methamphetamine and crack cocaine pipe distribution to PWUD in Mexico and Canada have found that these programs are associated with increased engagement with healthcare services and reduced pipe sharing [16–20]. Heroin smoking foils have been distributed in Germany, the UK, and the Netherlands to promote inhalation as an alternative to injection [23]. Despite the potential for RTIs to reduce harms associated with drug use, little has been published about the impact of safer smoking programs on drug injection behaviors or health outcomes among PWUD [24]. Additionally, few studies have evaluated whether pipe distribution may unintentionally increase risks associated with heroin smoking, including pneumonia, asthma exacerbation, and other pulmonary disease [25, 26].

A harm reduction service provider in Seattle, Washington, began offering heroin pipes to clients at a syringe service program (SSP) in 2019. This heroin pipe distribution program was developed at the request of SSP clients who expressed interest in heroin smoking as an alternative route of consumption that avoids difficulties in obtaining intravenous access and had a lower perceived risk of opioid overdose. We conducted a pretest–posttest quasi-experimental study to evaluate the impact of this novel harm reduction intervention on drug consumption behaviors and health outcomes among PWUH. We also aimed to measure potential harms associated with pipe distribution.

## Methods

### Study design

This quasi-experimental study used a one-group pretest–posttest design with multiple measurement timepoints. We conducted three weeklong pre-intervention observations at months 0, 1, and 2, and four weeklong post-intervention observations at months 4, 5, 6, and 9. The earliest pre-intervention timepoint was in March 2019, and latest post-intervention timepoint was in December 2019. The intervention was implemented at month 3 (June 2019). Three pre-intervention and four post-intervention timepoints were used to reduce threats to internal validity, including history (i.e., co-occurring events with the intervention that might account for observed changes in outcomes), instability (i.e., outcomes that are highly variable due to trends or cycles independent of the intervention), and maturation (i.e., changes in the characteristics of the community over time that confound the impact of the intervention).

### Study setting

This study was conducted at a single SSP site in Seattle, Washington, operated by The People’s Harm Reduction Alliance (PHRA), a community-based organization (CBO) that provides harm reduction services to PWUD. This SSP offers an array of harm reduction services to PWUD four days a week, including syringe distribution and disposal, naloxone training and distribution, and medication-assisted treatment for opioid use disorder. In the year prior to this study, 2.5 million syringes were distributed through this SSP in more than 17,000 client encounters. PHRA has also distributed crack cocaine pipes since 2010 and methamphetamine pipes since 2015.

### Participants

Study participants were recruited at the SSP site during service hours. Any PHRA client who accessed services during a pre-specified weeklong pre-intervention or post-intervention observation timepoint was offered the opportunity to be screened for eligibility to complete a survey. Eligibility criteria included being a PHRA client, ability to complete surveys in English, being 18 years old or older, and ability to provide informed consent. Participants could only complete one survey during each weeklong observation timepoint.

We intended to sample SSP clients during each weeklong observation timepoint as well as enroll a longitudinal cohort of SSP clients to be followed across timepoints. Therefore, before beginning a survey all participants were asked to provide a unique 6-character code comprised of their first and last initials, the first two letters of their mother’s first name, and the day of the month on which they were born. The code was used to prevent duplicate surveys from the same individual within a single timepoint and to track an individual’s responses across separate timepoints. Once enrolled, participants who opted to provide contact information were sent email or text message reminders prior to subsequent observation timepoints to promote retention.

### Intervention and comparator

The intervention was the implementation of a heroin pipe distribution program at an SSP. This program included the provision of pipes designed to smoke heroin and related education material. A total of 35 heroin pipes were available for distribution each day the SSP was in operation. Heroin pipes were distributed on a first-come first-served basis, and each client received a maximum of one pipe per day. Educational material described how the pipe could be used to smoke heroin as well as alerting users to the continued risk of opioid overdose (Additional file 3: Figure S1).

**Fig. 1:**
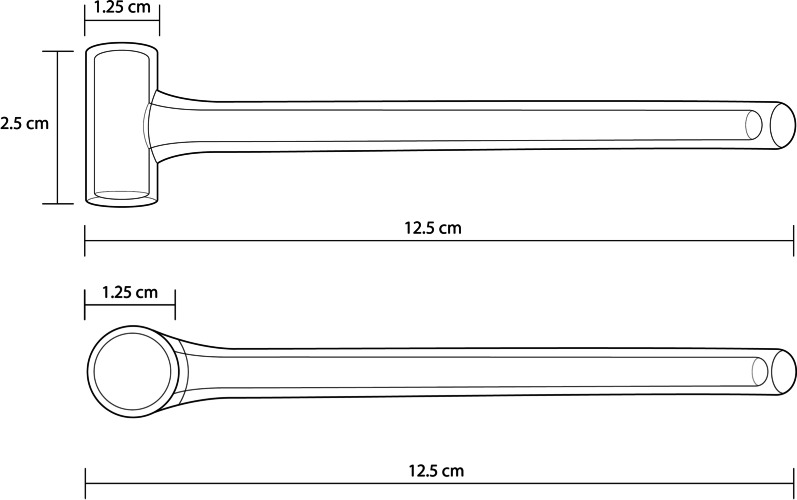
Heroin pipe used in this evaluation of heroin pipe distribution on drug consumption behaviors

The heroin pipes were collectively conceived and designed by PHRA staff, clients, and the Urban Survivors Union (USU), a grassroots coalition of PWUD. Focus groups with PHRA clients and USU members who inject and smoke heroin were conducted between May and August 2018 to evaluate a prototype design. Pipe shape and size were then refined based on participant feedback. The final version of the intervention pipe was manufactured by Pegasus Industrial Specialties Inc. (Fig. 1).

This study did not include a control group as all SSP participants were offered heroin pipes once the intervention was implemented. The comparator in this study was the period of time prior to implementation of heroin pipe distribution at the SSP. Besides the intervention, there were no other significant changes in SSP services between the pre- and post-intervention time periods.

### Data collection and assessments

Both pre- and post-intervention surveys collected self-reported information on the following domains: (1) sociodemographic characteristics, (2) drug consumption behaviors, (3) high-risk injection behaviors (e.g., syringe sharing, syringe reuse, public injection) (4) health outcomes associated with drug use (e.g., non-lethal opioid overdose, SSTI, hospitalization), (5) the HCV care continuum, and (6) social harms associated with crack cocaine or methamphetamine pipe distribution (e.g., arrest for pipe possession). Post-intervention surveys also collected data on utilization and perceived impact of the heroin pipe distribution program and potential harms associated with heroin pipe possession. Pre- and post-intervention survey forms are included in Additional file 1 and 2.

All surveys were administered verbally by volunteer research project staff. Survey forms were hosted on REDCap, a web application for designing and managing online surveys, and participant answers were input into online survey forms using a mobile device [27]. Draft versions of both pre- and post-intervention surveys were reviewed by SSP staff and volunteers prior to implementation. In consultation with our SSP partners, surveys were revised to make these instruments more culturally-competent, more relevant to local communities who use drugs, and less burdensome. Several alternative routes of heroin consumption, including snorting, rectal administration, and subcutaneous injection, were not included in surveys as SSP staff and volunteers reported these drug consumption behaviors were uncommon in the Seattle area.

### Sample size

As the intervention was a pilot project that had not been previously studied, an effect size could not be estimated, and therefore, no pre-specified sample size was set prior to recruitment. The final sample size was determined by the total number of eligible SSP clients who completed at least one survey.

### Outcomes

Primary outcomes were change in the proportion of SSP clients who exclusively injected heroin, exclusively smoked heroin, and both injected and smoked heroin in the past seven days as well as median number of weekly intravenous (IV) and intramuscular (IM) heroin injections between the pre- and post-intervention time periods. Secondary outcomes included changes in the proportion of SSP clients who used heroin by any route and reported syringe sharing, syringe reuse, and public injection in the past seven days as well as the proportion of SSP clients who used heroin and reported opiate overdose, SSTI, and hospitalization for any cause in the past 30 days.

### Sociodemographic measurements

Sociodemographic characteristics included age, gender, race, income, and housing status. Income was measured as a continuous variable and a binary variable (no income vs > $0 average monthly income). At each survey timepoint, participants were asked where they have been living most of the time during the past 30 days, and responses were categorized as housed (my own home/apartment or the home/apartment of friends/family) or unhoused (transitional housing/overnight shelter/on the street or in the car/other). Participants were also asked to report the gender of sexual partners during the past year, and we considered participants who reported being cisgender men and having sex with men in the prior year as men who have sex with men (MSM). We measured and reported MSM status in this study because MSM who inject drugs account for the majority of new HIV diagnosis among people who inject drugs in the Seattle area.

### Drug consumption and high-risk injection behavior measurements

All participants were asked if they had ever injected any drug and, if so, age when they first injected. We derived a measure of length of injection in years using the difference between the participant’s current age and age of first reported injection. Past month injection was assessed among those reporting lifetime injection. Syringe sharing, syringe reuse, and public injection in the past 30 days were assessed at each survey timepoint.

Participants were asked which drugs they had used in the past seven days (heroin by itself, meth by itself, cocaine/crack by itself, heroin and meth together (“goofball”), heroin and cocaine/crack together (“speedball”)) and the route of administration for each drug used (IV, IM, smoked). Participants were then asked how many times they used each drug via each reported route of administration in the past seven days. Those who reported heroin use were asked if they had tried to reduce their heroin use in the past 30 days. During the post-intervention period, surveys also asked participants if having access to the heroin pipe intervention made them smoke or inject heroin more frequently, less frequently, or had no impact on their frequency of use.

### Drug use-associated health outcome measurements

Surveys asked participants if they had an abscess, cellulitis, skin ulcerations, or been hospitalized in the past 30 days. Reports of an abscess, cellulitis, or a skin ulceration were combined into an SSTI measure. Participants who reported being hospitalized in the past 30 days were asked if they were diagnosed with infective endocarditis, an asthma or chronic obstructive pulmonary disorder exacerbation, pneumonia, or other lung infection during that hospitalization.

### Pipe use and social harms measurements

During the post-intervention period, participants were asked if they had smoked drugs in the past 30 days using the heroin pipe designed by PHRA. Participants who used a heroin pipe were asked what drugs they had used the heroin pipe to smoke, total number of heroin pipes they received, and number of times they were unable to obtain a heroin pipe from PHRA because of limited stock in the past 30 days. Participants were also asked if they had been arrested in the past 30 days and whether their arrest was a consequence of possessing a heroin pipe.

### Statistical analysis

We first described all unique survey respondents and compared sociodemographic characteristics and drug use behaviors of those who did and did not report using heroin during any survey timepoint. We then performed descriptive analyses comparing sociodemographic characteristics, drug consumption behaviors, high-risk injection behaviors, and health outcomes associated with drug use between the pre- and post-intervention periods for the whole sample as well as for only participants who used heroin. We also compared post-intervention survey responses between respondents who did and did not use a heroin pipe and by self-reported impact of heroin pipes on injection frequency.

Since the majority of participants only completed one survey, we collapsed survey timepoints into two time periods (i.e., pre-intervention and post-intervention) for comparisons. Furthermore, since only a small number of participants (14%) completed a survey in both the pre- and post-intervention periods, few of whom used heroin, all responses were treated as independent observations when comparing the pre- and post-intervention periods (Additional file 4: Table S1). For all binary variables, we considered the behavior as occurring during the pre-intervention period if it was reported on any pre-intervention survey, and the behavior as occurring during the post-intervention period if it was reported on any post-intervention survey. For continuous and categorical variables, if a respondent completed more than one pre-intervention survey, the latest response was used for the pre-intervention period, and if a respondent completed more than one post-intervention survey, the latest response was used for the post-intervention period. If participants reported different ages of first injection across different surveys, the oldest age was used. For variables with missing data, proportions were calculated using available data as the denominator.

Since all continuous variables were skewed, we compared continuous variables using Wilcoxon rank sum tests. We compared categorical variables using the Pearson chi-square tests and Fisher’s exact test for expected cell counts < 5. A *p* value < 0.05 was considered significant. Analyses were conducted in Stata 16.

### Ethics

The University of Washington Institutional Review Board approved this study (#00006488). Informed consent was obtained from all participants before beginning their first survey. Participants were compensated $5 USD after each completed survey.

## Results

A total of 694 unique respondents completed 957 surveys across the seven observation timepoints. The majority (59%, 408/694) of respondents used heroin in the past seven days during one or more survey timepoint. The median age of respondents who used heroin was 35 years (IQR 29–43). The majority identified as men (61%), white (63%), and currently unhoused (54%). Nearly half (46%) reported having no monthly income. Among respondents who used heroin, 90% reported lifetime injection drug use, including 79% and 25% who had consumed drugs through IV and IM injection in the past seven days during one or more timepoint, respectively, and 83% had smoked heroin in the past seven days during one or more timepoint. Sociodemographic characteristics and drug consumption behaviors of all respondents and respondents who used heroin are summarized in Table 1.

**Table 1:** Sociodemographic characteristics and drug use behaviors by reported heroin use in the past seven days

	Any heroin use (*n* = 408)		No heroin use (*n* = 286)	
	No. / Median	% / IQR	No. / Median	% / IQR
**Sociodemographic characteristics**				
Age ^#^				
Years	35	29–43	42	31–54
Gender ^#^				
Male	238	61%	215	81%
Female	145	37%	43	16%
Trans/Genderqueer/Non-binary	8	2%	8	3%
MSM status ^#^				
MSM	15	4%	24	9%
Race ^#^				
White/Caucasian	246	63%	122	46%
Black/African-American	23	6%	59	22%
Hispanic/Latino	17	4%	9	3%
Native American	23	6%	15	6%
Asian/Pacific Islander	15	4%	4	2%
Mixed Race	55	14%	47	18%
Other	12	3%	10	4%
Monthly income (USD) ^#^				
Median income	196	(0–1000)	192	(0–798)
No income	180	46%	121	46%
Housing status in past 30 days ^#^				
Housed	189	46%	109	38%
Unhoused	219	54%	175	62%
**Drug use behaviors**				
Drug consumption				
Exclusive heroin use	50	12%	0	N/A
Concurrent heroin and methamphetamine use				
(i.e., goofball)	222	54%	18	6%
Separate heroin and methamphetamine use	330	81%	N/A	N/A
Concurrent heroin and cocaine use (i.e., speedball)	45	11%	5	2%
Separate heroin and cocaine use	90	22%	N/A	N/A
Separate heroin, methamphetamine, and cocaine use	75	22%	N/A	N/A
Exclusive goofball use	0	0%	10	3%
Exclusive methamphetamine use	0	0%	166	58%
Exclusive cocaine use	0	0%	23	8%
Separate methamphetamine and cocaine use	75	18%	34	12%
Drug administration				
Lifetime history IDU ^#^	369	90%	177	63%
Number of years since first injection ^#^	8	(3–16)	14.5	(6–27)
Current IV injection (any drug)	322	79%	93	33%
Current IM injection (any drug)	100	25%	6	2%
Current smoking (any drug)	337	83%	214	75%

^#^ There were 43 missing values for income, 38 for age and sexual activity, 37 for gender and race, 3 for lifetime IDU history and past 30-day housing. There were also 25 missing values for number of years since first injected; 24 of these were due to missing age and 1 missing response of age first injected. Men who have sex with men (*MSM*). US dollars (*USD*). Injection drug use (*IDU*). Intravenous (*IV*). Intramuscular (*IM*)

### Respondents and surveys across the pre- and post-intervention periods

Of the 408 respondents who used heroin at any survey timepoint, 207 completed one or more pre-intervention survey, and 257 completed one or more post-intervention survey. Among respondents who used heroin, 79% (323/408) only completed one survey, and 21% (85/408) completed more than one survey, including 14% (56/408) who completed at least one survey during both the pre-intervention and post-intervention periods (Additional file 4: Table S1).

Of the total 957 surveys, 43% (415/957) and 57% (542/957) were completed during the three pre-intervention timepoints and four post-intervention timepoints, respectively. Multiple responses from a single respondent in a given period were collapsed, resulting in 790 records, of which 46% (360/790) were from the pre-intervention period and 54% (430/790) were from the post-intervention period. Heroin use was reported in the majority of pre-intervention (56%, 201/360) and post-intervention records (58%, 251/430). Measured sociodemographic characteristics did not differ significantly between the pre- and post-intervention periods (Additional file 4: Table S2).

### Heroin consumption behaviors among participants who used heroin across the pre- and post-intervention periods

**Fig. 2:**
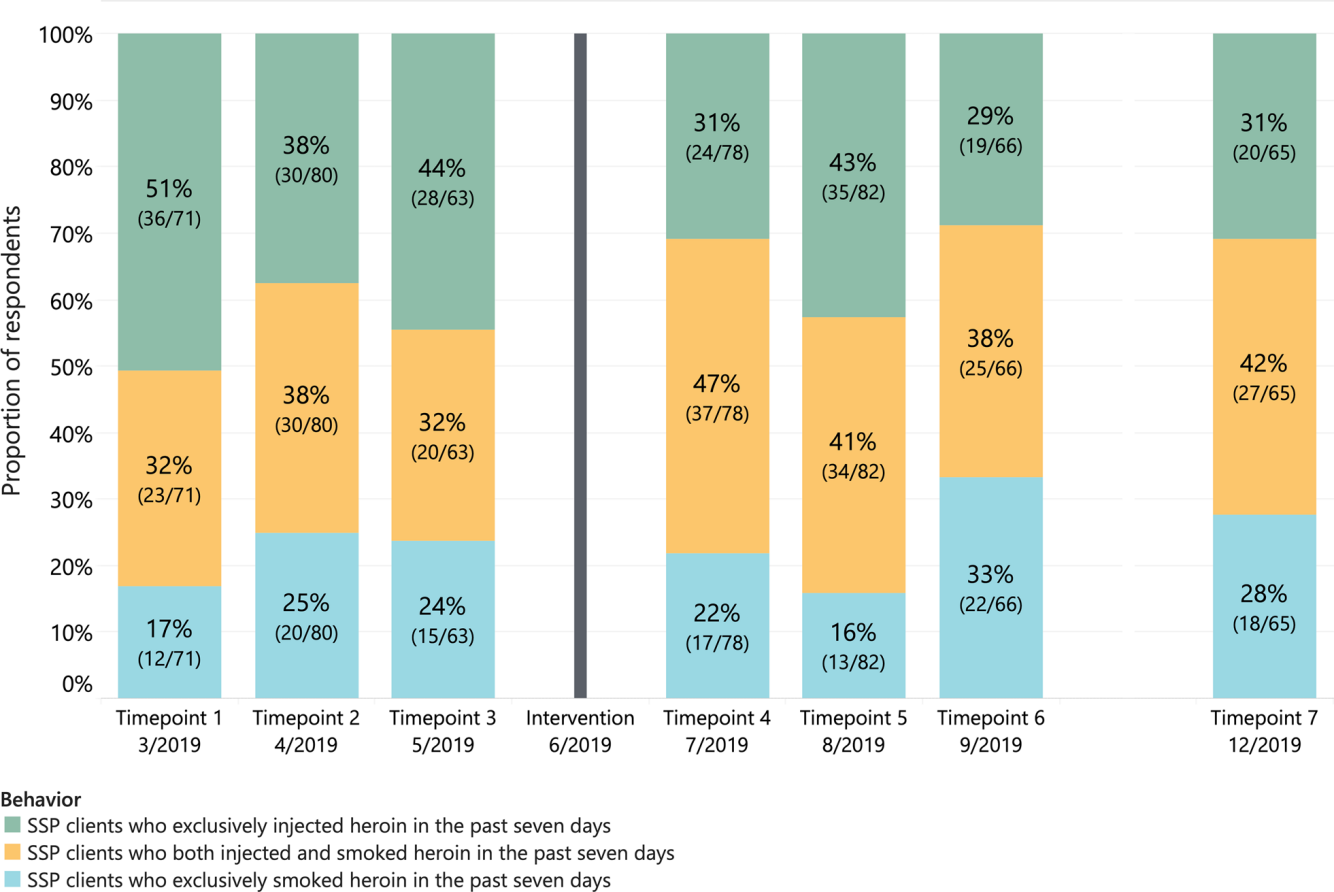
Proportion of respondents who injected, smoked, or injected and smoked heroin in the past week

The proportion of respondents who reported exclusively injecting heroin was significantly lower during the post-intervention than pre-intervention period (32%, 80/251 vs 43%, 86/201, *p* = 0.02), while the proportion of respondents who reported both injecting and smoking heroin was significantly higher during the post-intervention than pre-intervention period (45%, 113/251 vs 36%, 72/201, *p* = 0.048). The proportion of respondents who exclusively smoked heroin (*p* = 0.66), as well as median number of weekly IV injections (*p* = 0.15) and weekly IM injections (*p* = 0.16), did not change significantly comparing the periods before and after heroin pipes were distributed. Drug consumption behaviors across the pre- and post-intervention periods are presented in Table 2. The proportion of participants reporting exclusively injecting, exclusively smoking, or both injecting and smoking heroin are summarized in Fig. 2.

**Table 2:** Drug consumption behaviors and health outcomes before and after heroin pipe distribution among SSP clients

	Pre-intervention (*n* = 201)		Post-intervention (*n* = 251)		
	No. / Median	% / IQR	No. / Median	% /IQR	*p* value
**Heroin use behaviors in the past seven days**					
Exclusive IV and/or IM heroin use	86	43%	80	32%	0.02
Both IV and/or IM heroin use and heroin smoking	72	36%	113	45%	0.048
Exclusive heroin smoking	43	21%	58	23%	0.66
Median number times IV injection	20	4–30	20	7–30	0.16
Median number times IM injection	5	2–10	10	3–15	0.15
Median number times smoking heroin	5	2–15	5	2–14	0.58
**High-risk injection behaviors in the past 30 days**					
Syringe sharing ^#^	25	12%	23	9%	0.26
Syringe reuse ^#^	67	33%	63	25%	0.055
Public injection ^#^	103	51%	138	55%	0.43
**Drug use-associated health outcomes in the past 30 days**					
Self-reported non-lethal opioid overdose ^#^	15	7%	18	7%	0.92
SSTI (cellulitis and/or abscess) ^#^	58	29%	74	30%	0.86
Hospitalization (all cause) ^#^	32	16%	38	15%	0.83
Infective endocarditis (among those hospitalized) ^#^	0	0%	2	5%	0.50

^#^ There were 2 missing values for syringe sharing and reuse and 1 missing value for public injection, opioid overdose, SSTI, hospitalization, and endocarditis. Intravenous (*IV*). Intramuscular (*IM*). Skin and soft tissue infection (*SSTI*)

### High-risk injection behaviors and drug use-associated health outcomes across the pre- and post-intervention periods

The proportion of respondents who used heroin and reused syringes in the past seven days was lower after the implementation of heroin pipe distribution compared to the period prior to heroin pipe distribution (25%, 63/251 vs 33%, 67/201). This smaller portion of the overall sample who reported syringe reuse after implementation of heroin pipe distribution had borderline statistical significance (*p* = 0.055). The proportion of respondents who used heroin and shared syringes was lower during the post-intervention period than the pre-intervention period (9%, 23/251 vs 12%, 25/201); however, this difference in proportions was not statistically significant (*p* = 0.26). All other high-risk injection behaviors and drug use-associated health outcomes did not differ significantly between the pre- and post-intervention periods (Table 2).

### Heroin pipe uptake

A total of 251 respondents used heroin during the post-intervention period, of which 44% (110/251) used a heroin pipe from the SSP at least once within 30 days of completing a post-intervention survey. Among those who used a heroin pipe, 83% (91/110) used that pipe to smoke heroin, and 35% (38/108) had come to the SSP and been unable to procure a heroin pipe on one or more occasion because of limited stock. Compared to respondents who used heroin and did not use a heroin pipe, lower proportions of heroin pipe users exclusively injected heroin (11% vs 48%, *p* < 0.001), and higher proportions of heroin pipe users exclusively smoked heroin (32% vs 16%, *p* = 0.004) and both smoked and injected heroin (57% vs 35%, *p* = 0.001) in the past seven days. None of the respondents who had used a heroin pipe reported first-time injection drug use or being arrested for heroin pipe possession during the post-intervention period. Hospitalization for a pulmonary cause was not associated with using a heroin pipe (Table 3).

**Table 3:** Sociodemographic characteristics and study outcomes by heroin pipe use in the post-intervention period

	Used heroin pipe (*n* = 110)*§		Did not use heroin pipe (*n* = 141)*		
	No. / Average	% / IQR	No. / Average	% / IQR	*p* value
Obtained and used a heroin pipe from the SSP in the past 30 days	110	100%	N/A	N/A	N/A
Used that heroin pipe to smoke heroin (≥ 1 times)	91	83%	N/A	N/A	N/A
Unable to procure heroin pipe because out of stock (≥ 1 times) ^#^	38	35%	N/A	N/A	N/A
Median number of heroin pipes received per month at PHRA	2	1–3	N/A	N/A	N/A
**Sociodemographic characteristics**					
Age (years) ^#^	35	30–42	35	28–42	0.51
Gender ^#^					0.44
Male	63	60%	75	58%	
Female	41	39%	50	38%	
Trans/Genderqueer/Non-binary	1	1%	5	4%	
MSM status ^#^					1.00
MSM	3	3%	4	3%	
Race ^#^					0.08
White/Caucasian	60	57%	81	62%	
Black/African-American	9	9%	6	5%	
Hispanic/Latino	1	1%	7	5%	
Native American	3	3%	11	8%	
Asian/Pacific Islander	6	6%	4	3%	
Mixed Race	21	20%	16	12%	
Other	5	5%	5	4%	
Monthly income ^#^					
Median income (USD)	5	0–800	350	0–1200	0.17
No income	52	50%	56	43%	0.32
**Heroin consumption behaviors in the past seven days**					
Median number times IV injection	21	7–35	20	7–30	0.33
Median number times IM injection	10	2–14	7	3–20	0.49
Median number times smoking heroin	5	2–15	4	2–10	0.18
Exclusive IV and/or IM heroin use	12	11%	68	48%	< 0.01
Both IV and/or IM heroin use and heroin smoking	63	57%	50	35%	< 0.01
Exclusive heroin smoking	35	32%	23	16%	< 0.01
**Self-reported impact of heroin pipe distribution**					
Makes me smoke heroin more frequently ^#^	29	26%	17	12%	< 0.01
No impact on smoking frequency ^#^	76	69%	122	87%	
Makes me smoke heroin less frequently ^#^	5	5%	1	1%	
Makes me inject heroin more frequently ^#^	0	0%	0	0%	< 0.01
No impact on injection frequency ^#^	73	66%	116	83%	
Makes me inject heroin less frequently ^#^	37	34%	24	17%	
**High-risk injection behaviors in the past 30 days**					
Syringe sharing ^#^	8	7%	15	11%	0.36
Syringe reuse ^#^	21	19%	42	30%	0.05
Public injection ^#^	66	60%	72	51%	0.16
**Drug use-associated health outcomes in the past 30 days**					
Non-lethal opiate overdose ^#^	9	8%	9	16%	0.59
SSTI (cellulitis and/or abscess) ^#^	36	33%	38	27%	0.34
Hospitalization (all cause) ^#^	15	14%	23	16%	0.54
Infective endocarditis ^#^	2	2%	0	0%	0.15
**Potential harms associated with heroin pipe distribution in the past 30 days**					
Arrested for possession of a heroin pipe ^#^	0	0%	0	0%	N/A
Hospitalizations for pulmonary causes ^#^	4	4%	2	2%	0.19

^#^ There were 16 values missing for sociodemographic characteristics (age, gender, race, sexual orientation, income); two missing values for being unable to procure a pipe, arrest, and pulmonary hospitalizations; and one missing value for self-reported impact of pipe distribution, high-risk injection behaviors, and drug-related health outcomes. Syringe services program (*SSP*). The People’s Harm Reduction Alliance (*PHRA*). Men who have sex with men (*MSM*). US dollars (*USD*). Intravenous (*IV*). Intramuscular (*IM*). Skin and soft tissue infection (*SSTI*)

### Self-reported impact of heroin pipe distribution on frequency of heroin injection

Among the 250 respondents who used heroin in the post-intervention period and responded to how heroin pipe distribution affected their drug consumption behaviors, 24% (61/250) and 76% (189/250) reported the heroin pipe distribution program reduced and had no impact on the frequency of their heroin injection, respectively. None reported the heroin pipe program increased their injection frequency. Among the respondents who reported that heroin pipe distribution had reduced their injection frequency, a higher proportion both injected and smoked heroin compared to respondents who stated heroin pipe distribution had no impact on their injection frequency (59% vs 40%, *p* = 0.01). Fewer respondents who reported heroin pipe distribution had reduced their injection frequency exclusively smoked heroin in the post-intervention period compared to respondents who stated heroin pipe distribution had no impact on their injection frequency (13% vs 27%, *p* = 0.03) (Additional file 4: Table S3).

## Discussion

In this pilot pretest–posttest quasi-experimental study, we saw a lower proportion of SSP clients exclusively inject heroin and a higher proportion of SSP clients consume heroin through both injection and smoking after the implementation of a heroin pipe distribution program. The proportion of SSP clients who reported syringe reuse was also lower following the heroin pipe distribution intervention. We did not observe any difference in self-reported health outcomes associated with drug use between the pre- and post-intervention periods; however, the short follow-up period and small sample size of this pilot study may have contributed to this null finding. Our results suggest heroin pipe distribution may be a novel RTI that can be added to existing SSPs to further reduce harms associated with heroin use. This study also highlights the potential for public health service innovations to be developed by marginalized communities and the importance of placing PWUD in leadership positions in efforts to optimize harm reduction programming.

Despite the non-randomized design of this pilot study, several findings suggest heroin pipe distribution may have prompted changes in heroin consumption behaviors among PWUH. The proportion of SSP clients who exclusively injected heroin was lower by a quarter, while the proportion who both injected and smoked heroin was higher by over a quarter after heroin pipe distribution began. Twenty-four percent of respondents who used heroin reported heroin pipe distribution had reduced their heroin injection. Higher proportions of SSP clients who received heroin pipes exclusively smoked heroin or both smoked and injected heroin compared to SSP clients who did not receive a heroin pipe. We are unaware of any prior published research investigating heroin pipes as an RTI; however, pre–post-analyses examining foil distribution at SSPs in Europe found similar changes in drug consumption behaviors, with up to 85% of SSP clients having used foil to inhale rather than inject heroin on at least one occasion [23, 28]. Our non-randomized study design cannot control for confounding and prevents firm conclusions as to whether this observed shift from injection to smoking can be attributed to the intervention. Additionally, only 14% of respondents who used heroin completed surveys during both the pre- and post-intervention periods, and thus, outcomes may have been impacted by changes in the SSP client population across time periods. Further experimental research is needed to clarify the causal relationship between heroin pipe distribution and reductions in heroin injection. Study designs that are randomized by individual may be complicated by heroin pipe sharing across intervention and control groups. Cluster randomization may better control for contamination given extensive social networks and resource exchange among PWUD [29].

We found that the proportion of SSP clients who reused syringes was lower in the post-intervention period compared to the pre-intervention period, and this difference in proportions had borderline statistical significance. Otherwise, we did not observe any significant differences in high-risk injection behaviors or health outcomes associated with drug use after implementation of heroin pipe distribution. Several reasons may explain this null finding. The proportion of SSP clients who exclusively smoked heroin and the median injection frequency did not change with distribution of heroin pipes, suggesting the primary behavioral change after the onset of the intervention was a shift from exclusive injection to mixed injection-smoking consumption. PWUH who employ mixed heroin consumption behaviors may still engage in high-risk practices when injecting. A more complete and sustained transition from heroin injection to smoking may be necessary before reductions in SSTI, blood-borne infections, and overdose become evident. Additionally, only 36% of all respondents reported using heroin after heroin pipe distribution began, of whom less than half used the heroin pipe to smoke heroin within 30 days of taking a post-intervention survey. While SSTI and non-lethal opioid overdose are not rare outcomes among PWUH (occurring in roughly 25% and 5% of SSP clients per month in this study, respectively), the small number of SSP clients exposed to the intervention may have contributed to our null results. Study designs with longer follow-up periods and larger sample sizes are necessary to evaluate for less common health outcomes, including infective endocarditis as well as incident HIV and HCV infection. Additionally, there are several high-risk drug consumption behaviors (e.g., consuming opioids alone) and health outcomes associated with heroin injection and inhalation (e.g., bacteremia, osteomyelitis, toxic leukoencephalopathy) that were not measured in this pilot study [30, 31]. High-risk smoking behaviors, such as pipe sharing and injury to lips from using low-quality smoking equipment, may also be prevented through pipe distribution programs, and these programs may facilitate linkage to other harm reduction services, such as medication-assisted treatment [16–20]. These outcomes were not measured by our survey instrument. Future studies should include a wider range of outcome measures to more fully assess the full impact of heroin pipe distribution on the health of PWUH.

Most respondents who used heroin during the post-intervention period did not receive a heroin pipe. Many programmatic and client factors may have limited exposure to the intervention. Heroin pipes were offered on a first-come first-served basis, and clients that visited the SSP later in the day were less likely to receive a pipe due to limited stock. A third of pipe recipients reported heroin pipes had been unavailable on at least one SSP visit. Our survey did not ask respondents whether they were aware of the new heroin pipe distribution program, and the program was primarily advertised informally by word of mouth. Therefore, SSP clients may not have been exposed to the intervention because they did not know to ask for a pipe. Beyond programmatic limitations, certain SSP clients may have been more likely to accept a heroin pipe when available. Exclusively injecting heroin was nearly five times more common among those who did not receive a heroin pipe than among pipe recipients, suggesting that SSP clients habituated to heroin injection may have been more likely to refuse a pipe when offered. However, 15% of respondents who exclusively injected heroin still obtained a heroin pipe on at least one occasion, which may indicate willingness to try new consumption methods. Different chemical forms of heroin are available in the USA, including hydrochloride salt and free base forms of heroin. Compared to its free base form, heroin hydrochloride salt is much less volatile and requires higher temperatures for volatilization [32]. Some PWUH in this study may have chosen to avoid heroin pipes because they typically use heroin hydrochloride salt which is less easily smoked [33]. By containing heroin in a small area enclosed by Pyrex, the heroin pipe piloted in this study may allow PWUH to more reliably achieve the higher temperatures needed to effectively volatilize heroin hydrochloride salt and achieve the desired pharmacological effects compared to other inhalation methods (e.g., foil). Future quantitative and qualitative research is needed to better understand differences in heroin pipe uptake among PWUH, assess feasibility, and optimize the intervention’s impact on high-risk drug consumption behaviors.

We evaluated several theoretical risks associated with provision of heroin pipes through SSPs in this pilot study and found no evidence to support these concerns. Distribution of heroin pipes through an SSP could introduce heroin smokers to injection supplies, inadvertently promoting a transition from heroin smoking to injection. No heroin pipe recipients reported first-time injection during the study. Heroin smoking is associated with pulmonary disease, including potentially life-threatening asthma and chronic obstructive pulmonary disease (COPD) exacerbations [25], and increased heroin smoking through heroin pipe distribution could lead to worsened pulmonary complications for PWUH. However, self-reported hospitalizations for pulmonary causes did not significantly differ before and after heroin pipe distribution or between respondents who used and did not use heroin pipes obtained from the SSP. In certain jurisdictions, including Washington State, legal protections for possession of injection supplies do not extend to heroin pipes, and pipe distribution may place PWUH at higher risk of arrest. No SSP clients reported being arrested or detained for heroin pipe possession during the post-intervention period.

Our pilot study has several limitations. The absence of randomization in our pretest–posttest design does not allow us to control for confounding of unmeasured variables and prevents us from establishing causation between the intervention and outcomes. We conducted seven separate observation timepoints to reduce threats to internal validity and minimize selection bias. However, with only three pretest observations we were unable to employ the regression-based approach commonly used in interrupted time series analysis, limiting our ability to control for possible external time-varying effects or autocorrelation which may account for changes in outcomes over time. SSP clients were recruited by convenience sampling during each observation timepoint and few respondents completed surveys in two or more time periods. While changes in outcomes may have been driven by changes in the sampled population rather than changes in drug consumption behaviors of individuals, measured sociodemographic characteristics did not differ significantly between respondents who completed surveys in the pre- and post-intervention periods. We originally intended to recruit a longitudinal cohort of SSP clients and analyze changes in drug consumption behaviors of unique SSP clients over time; however, only 14% of respondents were surveyed in both the pre- and post-intervention periods. Our experience suggests text message or email reminders and small cash incentives may not be sufficient to prevent loss-to-follow-up among PWUD, and innovative strategies are needed to improve PWUD retention in longitudinal research projects. Finally, outcomes were measured by self-report, which introduce the possibility of misclassification due to recall, information, and social desirability biases. However, unless referring to lifetime use, respondents were only asked to report on behaviors occurring within the past 30 or seven days to minimize recall bias, and surveys were administered by longtime SSP volunteer staff to make respondents feel as comfortable as possible when disclosing sensitive information about their drug use and health behaviors.

## Conclusions

Our pilot study suggests heroin pipe distribution at an SSP may be a novel RTI to change drug consumption behaviors and reduce harms associated with heroin injection. Future research is needed to expand on our findings, including larger randomized trials to evaluate whether changes in drug consumption behavior prompted by pipe distribution improve downstream health outcomes. Qualitative and mixed-methods investigations would be helpful to assess acceptability and optimize delivery of heroin pipe distribution across different settings as well as evaluate the impact of this intervention on the frequency and quantity of opioids used. Access to heroin pipes may delay initiation of heroin injection by providing PWUH with an effective alternative to heroin injection. Studies outside of SSPs, where most PWUH have already begun injecting heroin, could assess whether heroin pipe distribution delays or prevents injection among people who recently started using heroin. People who inject methamphetamine and cocaine are also at risk for many of the same poor health outcomes as PWUH, and future investigations could be expanded to include methamphetamine and crack cocaine pipe distribution. Intranasal and rectal heroin administration are alternative routes of consumption which may have fewer risks compared to injection and inhalation. Other novel RTIs, including provision of supplies for heroin snorting or booty bumping, deserve further attention. Additionally, this study was conducted prior to the COVID-19 pandemic. Pipe distribution programs may prevent pipe sharing, and thereby COVID-19 transmission, among PWUD. The impact of safer smoking programs on COVID-19 outcomes should be incorporated into future research.

Future research should pay particular attention to the potential impact of pipe distribution on fentanyl use. Both fentanyl injection and inhalation have become increasingly central to opioid use patterns and overdose risk in the USA [34]. Trends away from opioid injection and toward fentanyl inhalation have been observed among PWUH in some regions of the USA, potentially driven by ease of use, feelings of improved health, and reduced stigma with fentanyl smoking [35]. Given many PWUH find fentanyl to be more easily smoked than heroin, pipe distribution may accelerate the transition from opioid injection to fentanyl smoking and thereby reduce risk of injection-related infections. When this pilot study was conceived, the growing extent of fentanyl use in the Seattle area was not fully appreciated, and consequently, we did not ask respondents to report fentanyl injection or inhalation in this pilot study. Future evaluations of pipe distribution should measure changes in both heroin and fentanyl consumption behaviors. Pipes may need to be redesigned to meet the needs of people who use fentanyl, and evaluations of RTIs targeting fentanyl use should pay particular attention to the impact on risk of opioid overdose.

Finally, this project represents the culmination of a new harm reduction service conceived, designed, and implemented by members of the drug using community. The PWUD leaders who guided this project through development deserve credit for this innovation. Peer participation and leadership is increasingly recognized as essential to finding creative solutions that effectively and ethically meet the needs of PWUD. Space should be made for PWUD leadership in future evaluations of heroin pipe distribution and other harm reduction programming.

## Supplementary Information

**Additional file 1:** Post-intervention survey.

**Additional file 2:** Pre-intervention survey.

**Additional file 3:** Educational materials distributed to SSP clients describing how the pipe could be used to smoke heroin.

**Additional file 4:** Supplementary tables.

## Data Availability

The datasets generated and/or analyzed during the current study are available from the corresponding author on reasonable request.

## Abbreviations

PWUH: People who use heroin
HIV: Human immunodeficiency virus
HCV: Hepatitis C virus
SSTI: Skin and soft tissue infections
RTI: Route transition intervention
PWUD: People who use drugs
SSP: Syringe service program
PHRA: The People’s Harm Reduction Alliance
CBO: Community-based organization
USU: Urban Survivors Union
IV: Intravenous
IM: Intramuscular
MSM: Men who have sex with men
COPD: Chronic obstructive pulmonary disease
