# Supplementary material for: Heroin pipe distribution to reduce high-risk drug consumption behaviors among people who use heroin: a pilot quasi-experimental study
Source: Harm Reduct J. 2022 Sep 22;19:103. doi: 10.1186/s12954-022-00685-7 (PMC9493152; doi:10.1186/s12954-022-00685-7)
Supplement: Supplementary file 2 — Additional file 2: Pre-intervention survey. [file 12954_2022_685_MOESM2_ESM.pdf]

Pipe Study - Pre-Intervention #3

Participant identifiers (This information will not be shared with anyone and will be stored in a secure database. We will use this information to link past and future survey responses, so please provide information that will not change over time.)

- What are the initials of your first and last name? (e.g. John Doe = "JD")
- What are the first two letters of your mother's name? (e.g. (Jane Doe = "JA")
- On what day of the month were you born? (e.g. 04 December 1983 = "04")

____

Is this the participant's first survey?

◯ Yes
◯ No

Have you already taken a survey at PHRA in the past week?

◯ Yes
◯ No

Sociodemographic characteristics

Question	Response
How do you identify your gender?	◯ Male<br>◯ Female<br>◯ Trans man<br>◯ Trans woman<br>◯ Genderqueer / Non-binary<br>◯ Unsure / Other
Do you identify as transgender?	◯ Yes<br>◯ No
How old are you?	__________________________
What do you consider your race? [Select all that apply]	☐ White / Caucasian<br>☐ Black / African-American<br>☐ Foreign-born African<br>☐ Asian / Pacific Islander<br>☐ Native American<br>☐ Hispanic / Latino<br>☐ Other
Please specify	__________________________
What is your average monthly income?	__________________________
What zip code or neighborhood do you live in?	__________________________
In the past month (30 days), where have you been living most of the time?	◯ My own home / apartment<br>◯ The home / apartment of friends / family (e.g. couch surfing, not on the lease)<br>◯ Transitional housing (e.g. halfway house)<br>◯ Overnight shelter<br>◯ On the street or in a car<br>◯ Other
Please specify	__________________________
Who do you have sex with? Sex includes any oral, anal, or vaginal sex. [Select all that apply]	☐ Men<br>☐ Women<br>☐ Trans men<br>☐ Trans women<br>☐ Non-binary / Genderqueer<br>☐ I don't have sex

Drug use behaviors

Have you ever injected drugs?
◯ Yes
◯ No

How old were you when you first injected?
____

Have you injected any drug in the past month?
◯ Yes
◯ No

In the following table, please indicate which of the following drugs you have used in the past week (seven days) and how you have used them:

	Heroin by itself	Meth by itself	Cocaine / crack by itself	Heroin and meth together ("goofball")	Heroin and cocaine / crack together ("speedball")
Injected into a vein	☐	☐	☐	☐	☐
Injected into a muscle	☐	☐	☐	☐	☐
Smoked	☐	☐	☐	☐	☐

Injecting into a Vein

How many times have you injected heroin into a vein in the past week (seven days)? __________________________________

How many times have you injected meth into a vein in the past week (seven days)? __________________________________

How many times have you injected crack / cocaine into a vein in the past week (seven days)? __________________________________

How many times have you injected heroin and meth together ("goofball") into a vein in the past week (seven days)? __________________________________

How many times have you injected heroin and cocaine / crack together ("speedball") into a vein in the past week (seven days)? __________________________________

Injecting into a Muscle

How many times have you injected heroin into a muscle in the past week (seven days)?	________________________
How many times have you injected meth into a muscle in the past week (seven days)?	________________________
How many times have you injected crack / cocaine into a muscle in the past week (seven days)?	________________________
How many times have you injected heroin and meth together ("goofball") into a muscle in the past week (seven days)?	________________________
How many times have you injected heroin and cocaine / crack together ("speedball") into a muscle in the past week (seven days)?	________________________

Smoked

How many times have you smoked heroin in the past week (seven days)?	________________
How many times have you smoked meth in the past week (seven days)?	________________
How many times have you smoked crack / cocaine in the past week (seven days)?	________________
How many times have you smoked heroin and meth together ("goofball") in the past week (seven days)?	________________
How many times have you smoked heroin and cocaine / crack together ("speedball") in the past week (seven days)?	________________

Harm reduction behaviors

When was the first time you came to PHRA?	◯ Today<br>◯ Within the past month<br>◯ Within the past 3 months<br>◯ Within the past 6 months<br>◯ Within the past year<br>◯ Over a year ago
In the past month (30 days), how many times have you come to PHRA to receive services?	______________________________
In the past month (30 days), have you received new syringes through any harm reduction / syringe exchange program?	◯ Yes<br>◯ No
How many of those syringes did you receive here at PHRA?	◯ All<br>◯ Most<br>◯ About half<br>◯ Less than half<br>◯ None
In the past month (30 days), on average how many times did you use each syringe before getting rid of it?	______________________________
In the past month (30 days), how many times have you shared a syringe, either you used it first or someone else did?	______________________________
In the past month (30 days), have you injected in a public space?	◯ Yes<br>◯ No
In the past month (30 days), have you or someone else used your cooker more than once?	◯ Yes<br>◯ No
If yes, did you: (check all that apply)	☐ Use your cooker more than once<br>☐ Share your cooker (either you used it first or someone else did)
In the past month (30 days), have you or someone else used your cotton more than once?	◯ Yes<br>◯ No
If yes, did you: (check all that apply)	☐ Use your cotton more than once<br>☐ Share your cotton (either you used it first or someone else did)
In the past month (30 days), have you gotten Naloxone/Narcan anywhere?	◯ Yes<br>◯ No
In the past month (30 days), did you get Naloxone/Narcan here at PHRA?	◯ Yes<br>◯ No
In the past month (30 days), have you used that Naloxone/Narcan?	◯ Yes<br>◯ No

In the past month (30 days), how many times have you come to PHRA for Naloxone / Narcan and not received it because they were out of stock? __________________________________

Meth Pipes

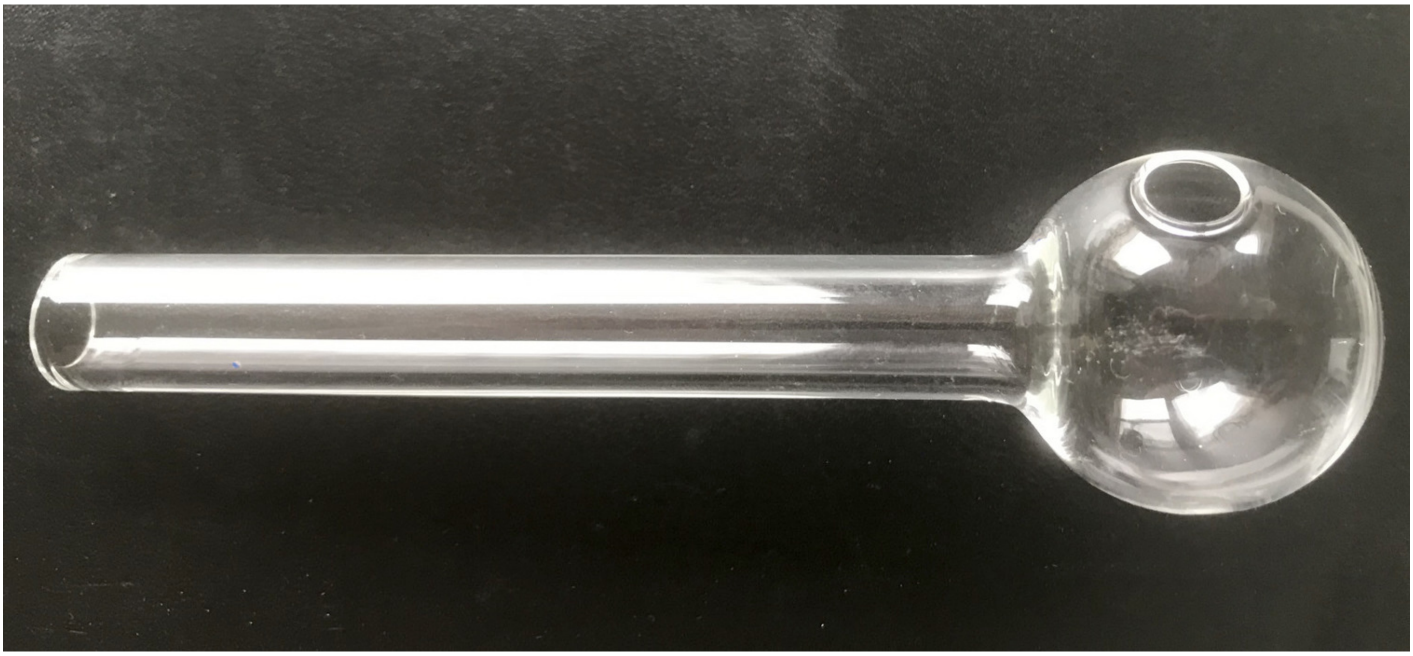

In the past month (30 days), have you smoked drugs using this type of pipe (i.e. bubble, meth pipe)?

◯ Yes
◯ No

In the past month (30 days), which of the following drugs have you smoked using this type of pipe (i.e. meth pipe) (select all that apply)?

☐ Meth
☐ Crack / cocaine
☐ Heroin / opiates
☐ Heroin and meth together ("goofball")
☐ Heroin and crack / cocaine together ("speedball")
☐ Other

Please specify which other drugs you have smoked

____

In the past month (30 days), where have you gotten this type of pipe (i.e. meth pipe) (select all that apply)?

☐ Here at PHRA
☐ From another person (gifted, traded, or purchased)
☐ At a store
☐ From another harm reduction / public health organization
☐ Other

Please specify where else you have received this type of pipe

____

In the past month (30 days), how many of these pipes (i.e. meth pipe) have you gotten here at PHRA?

____

In the past month (30 days), how many times have you come to PHRA for this type of pipe (i.e. meth pipe) and not received one because they were out of stock?

____

How does the distribution of this type of pipe (i.e. meth pipe) at this exchange affect the frequency of your meth smoking?

◯ Makes me smoke meth more frequently
◯ No impact
◯ Makes me smoke meth less frequently

How does the distribution of this type of pipe (i.e. meth pipe) at this exchange affect the frequency of your meth injecting?	◯ Makes me inject meth more frequently<br>◯ No impact<br>◯ Makes me inject meth less frequently

Crack Pipes

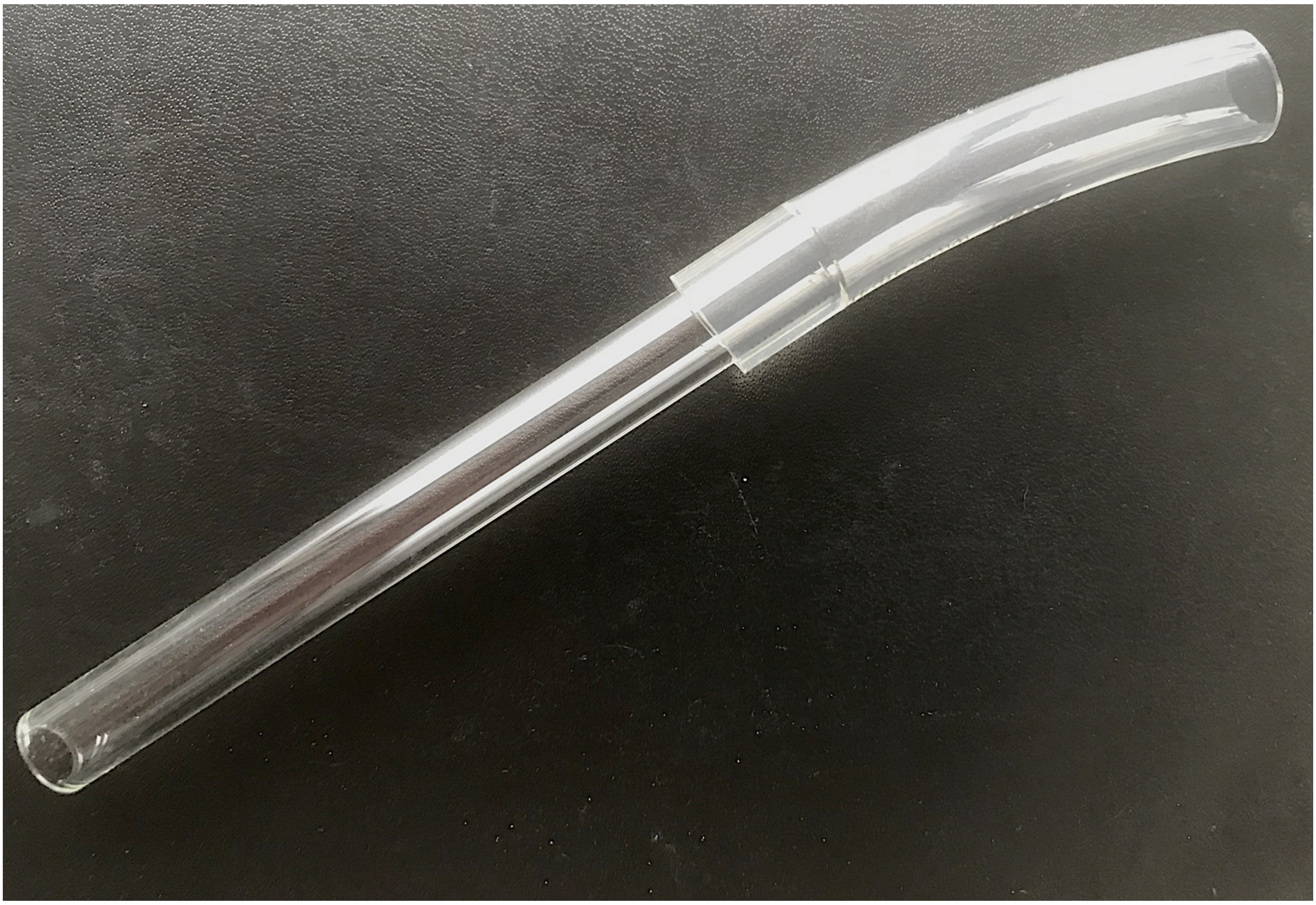

In the past month (30 days), have you smoked drugs using this type of pipe (i.e. stem, crack pipe)?	○ Yes<br>○ No
In the past month (30 days), which of the following drugs have you smoked using this type of pipe (i.e. crack pipe) (select all that apply)?	☐ Meth<br>☐ Crack / cocaine<br>☐ Heroin / opiates<br>☐ Heroin and meth together ("goofball")<br>☐ Heroin and crack / cocaine together ("speedball")<br>☐ Other
Please specify which other drugs you have smoked	__________________________
In the past month (30 days), where have you gotten this type of pipe (i.e. crack pipe) (select all that apply)?	☐ Here at PHRA<br>☐ From another person (gifted, traded, or purchased)<br>☐ At a store<br>☐ From another harm reduction / public health organization<br>☐ Other
Please specify where else you have received this type of pipe	__________________________
In the past month (30 days), how many of these pipes (i.e. crack pipe) have you gotten here at PHRA?	__________________________

In the past month (30 days), how many times have you come to PHRA for this type of pipe (i.e. crack pipe) and not received one because they were out of stock?	________________________
How does the distribution of this type of pipe (i.e. crack pipe) at this exchange affect the frequency of your crack smoking?	◯ Makes me smoke crack more frequently<br>◯ No impact<br>◯ Makes me smoke crack less frequently
How does the distribution of this type of pipe (i.e. crack pipe) at this exchange affect the frequency of your crack injecting?	◯ Makes me inject crack more frequently<br>◯ No impact<br>◯ Makes me inject crack less frequently
How would you describe your interest in receiving pipes here at PHRA specifically designed to smoke heroin?	◯ Very interested<br>◯ Somewhat interested<br>◯ Neither interested nor disinterested<br>◯ Somewhat disinterested<br>◯ Very disinterested
If we began distributing pipes here at PHRA specifically designed to smoke heroin, how likely would you be to use them to smoke heroin?	◯ Very likely<br>◯ Somewhat likely<br>◯ Uncertain<br>◯ Somewhat unlikely<br>◯ Very unlikely

Health outcomes

In the past month (30 days), have you experienced an opiate overdose that left you unconscious, required the administration of Naloxone/Narcan, or resulted in the paramedics being called?
- ◯ Yes
- ◯ No

In the past month (30 days), how many opiate overdoses have you experienced?
____

In the past month (30 days), how had you used heroin / opiates when you overdosed? (select all that apply)
- ☐ Injected into a vein
- ☐ Injected into a muscle
- ☐ Smoked

Which of the following drugs, if any, did you consume in addition to heroin / opiates during any of the overdoses you experienced in the past month (30 days)? (select all that apply)
- ☐ Alcohol
- ☐ Benzodiazepines (Benzos)
- ☐ Methamphetamine
- ☐ Cocaine / crack
- ☐ Other

Please specify which other drugs you used in addition to opiates during your overdose
____

In the past month (30 days), have you witnessed an overdose that left someone else unconscious, required the administration of Naloxone/Narcan, or resulted in the paramedics being called?
- ◯ Yes
- ◯ No

In the past month (30 days), how many overdoses have you witnessed?
____

In the past month (30 days), how many of the overdoses you witnessed were fatal?
____

In the past month (30 days) have you had a heart attack, stroke, seizure, intense overheating or really extreme, sudden psychosis because of stimulant use (this is also called overamping)?
- ◯ Yes
- ◯ No

In the past month (30 days), how many times have you overamped?
____

In the past month (30 days), have you had cellulitis (i.e. a skin infection with redness, swelling, warmth, and/or pain) or skin ulcerations?
- ◯ Yes
- ◯ No

In the past month (30 days), have you had an abscess?
- ◯ Yes
- ◯ No

In the past month (30 days), have you been hospitalized?
- ◯ Yes
- ◯ No

During any hospitalization in the past month (30 days), were you diagnosed as having cellulitis or a skin/soft tissue infection?
- ◯ Yes
- ◯ No

During any hospitalization in the past month (30 days), were you diagnosed as having endocarditis or an infection involving the valves of your heart?	◯ Yes<br>◯ No
During any hospitalization in the past month (30 days), were you diagnosed as having an asthma exacerbation, COPD exacerbation, pneumonia, or other lung infection?	◯ Yes<br>◯ No
In the past month (30 days), how often have you felt that your drug use is too chaotic or out of control?	◯ All the time<br>◯ Most of the time<br>◯ Sometimes<br>◯ Occasionally<br>◯ Never
In the past month (30 days), have you tried to reduce your heroin / opiate use?	◯ Yes<br>◯ No<br>◯ N/A: I haven't done heroin in the past 30 days
In the past month (30 days), what methods have you used to reduce your heroin / opiate use?	☐ Used Methadone<br>☐ Used Suboxone / Buprenorphine<br>☐ Received substance use counseling<br>☐ Transitioned to using other prohibited drugs<br>☐ Injected heroin / opiates less frequently<br>☐ Smoked heroin / opiates less frequently<br>☐ Other (please specify)
Please specify which drugs you have used to reduce your heroin use	__________________________________
Please specify how you have tried to reduce your heroin use	__________________________________
In the past month (30 days), have you tried to reduce your methamphetamine use?	◯ Yes<br>◯ No<br>◯ N/A: I haven't used methamphetamine in the past 30 days
In the past month (30 days), what methods have you used to reduce your methamphetamine use?	☐ Received substance use counseling<br>☐ Transitioned to using other prohibited drugs<br>☐ Injected methamphetamine less frequently<br>☐ Smoked methamphetamine less frequently<br>☐ Other (please specify)
Please specify which drugs you have used to reduce your meth use	__________________________________
Please specify how you have tried to reduce your meth use	__________________________________

Depression and Anxiety

How concerned are you about depression?

◯ Extremely concerned
◯ Moderately concerned
◯ Somewhat concerned
◯ Slightly concerned
◯ Not at all concerned

How concerned are you about anxiety?

◯ Extremely concerned
◯ Moderately concerned
◯ Somewhat concerned
◯ Slightly concerned
◯ Not at all concerned

Social harms

In the past month (30 days), have you been arrested?
Yes
No

◯ Yes
◯ No

In the past month (30 days), have you been in jail or prison for more than 24 hours?

◯ Yes
◯ No

Was any arrest or incarceration in the past month (30 days) related to possessing drug use paraphernalia?

◯ Yes
◯ No

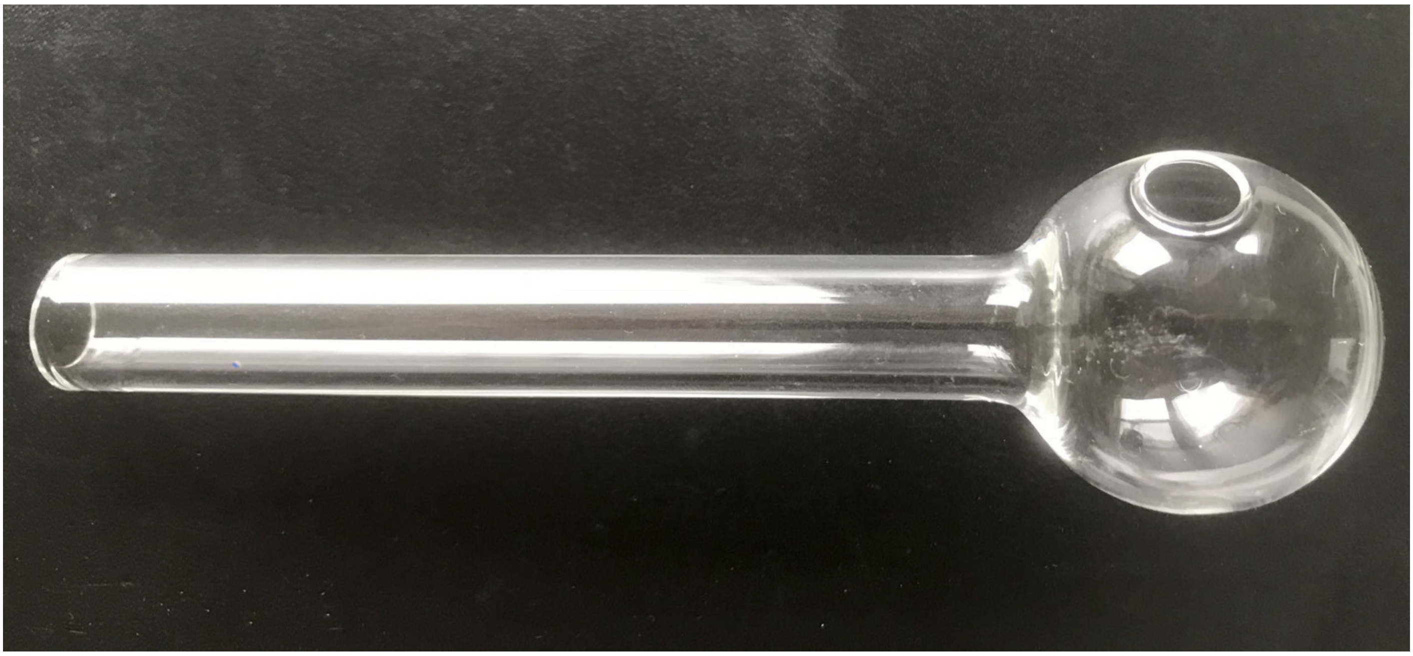

Was any arrest or incarceration in the past month (30 days) related to possessing this type of pipe (i.e. bubble, meth pipe)?

◯ Yes
◯ No

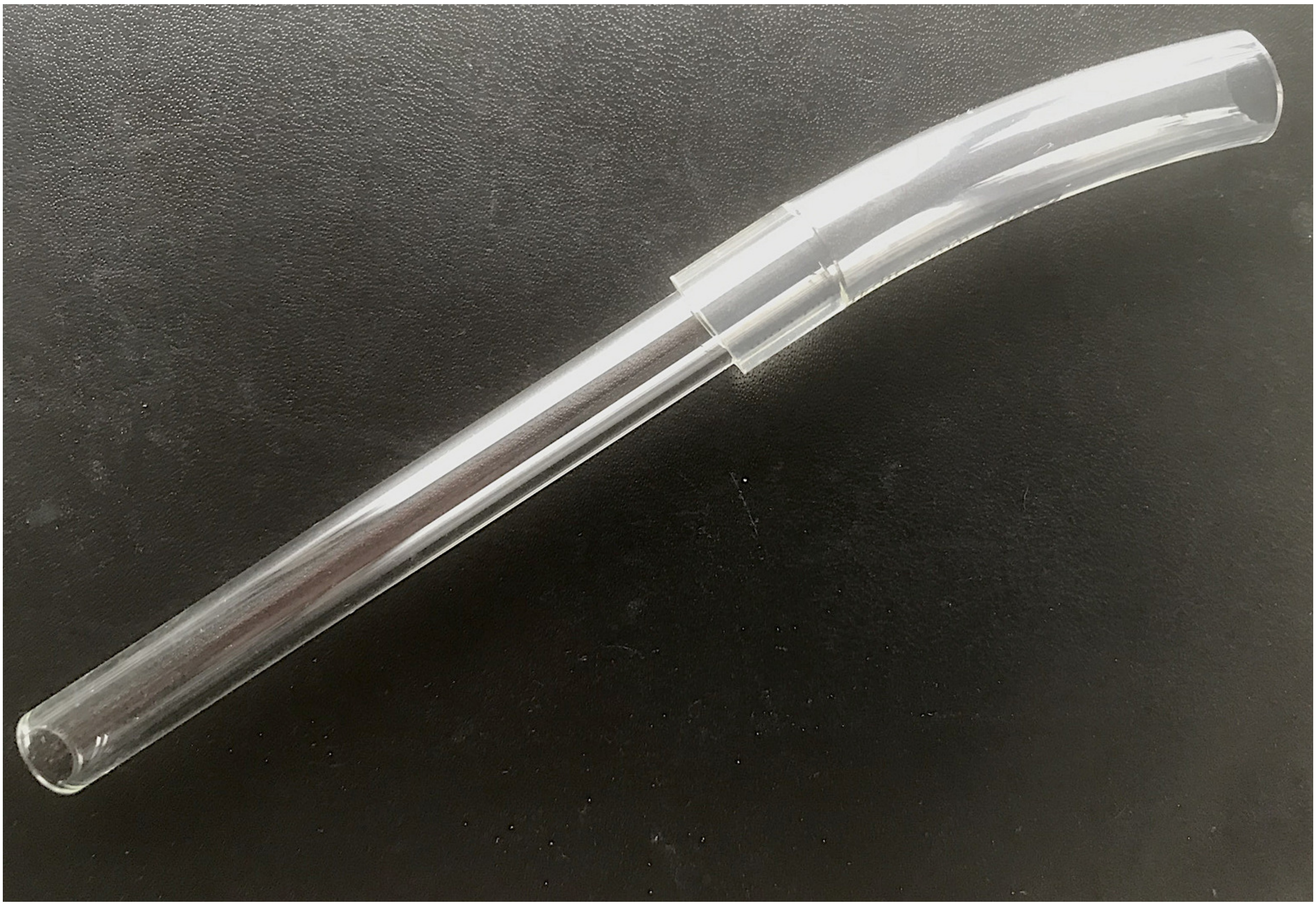

Was any arrest or incarceration in the past month (30 days) related to possessing this type of pipe (i.e. stem, crack pipe)?	◯ Yes<br>◯ No
Since we started doing surveys at PHRA in March 2019 two months ago have you been in jail/prison, inpatient treatment, the hospital, or not in Seattle for a period of 30 days or more?	◯ Yes<br>◯ No
If yes, where were you?	☐ Jail/prison<br>☐ Inpatient treatment<br>☐ Hospitalization<br>☐ Traveling outside of Seattle<br>☐ Other (please specify)
Please specify why you were not in Seattle for 30 days or more since we started doing surveys at PHRA in March 2019.	__________________________________

Hepatitis C care continuum

Have you ever been tested for hepatitis C?	◯ Yes<br>◯ No
Have you ever been tested for hepatitis C here at PHRA?	◯ Yes<br>◯ No
Have you been tested for hepatitis C in the past year?	◯ Yes<br>◯ No
Have you been tested for hepatitis C in the past year here at PHRA?	◯ Yes<br>◯ No
Did you receive the results of your last hepatitis C test?	◯ Yes<br>◯ No
Have you ever been diagnosed with having hepatitis C?	◯ Yes<br>◯ No
What year did you receive your hepatitis C diagnosis?	______________________
Have you ever been told that you cleared your hepatitis C infection without treatment?	◯ Yes<br>◯ No
Have you ever sought treatment for hepatitis C from a doctor?	◯ Yes<br>◯ No
Has a doctor ever told you that you do not qualify for hepatitis C treatment because you are currently using drugs?	◯ Yes<br>◯ No
Have you heard that there are new oral medications (direct acting agents, DAAs) that can effectively cure hepatitis in 2-3 months?	◯ Yes<br>◯ No
Have you ever received treatment for hepatitis C?	◯ Yes<br>◯ No
Was your hepatitis C cured?	◯ Yes<br>◯ No
Notes<br><br>This section is for the interviewer to include any notes about the interview that they feel are relevant to the data collected. No identifiers may be recorded here.	___________________________
