# Supplementary figures and images for: Heroin pipe distribution to reduce high-risk drug consumption behaviors among people who use heroin: a pilot quasi-experimental study
Source: Harm Reduct J. 2022 Sep 22;19:103. doi: 10.1186/s12954-022-00685-7 (PMC9493152; doi:10.1186/s12954-022-00685-7)

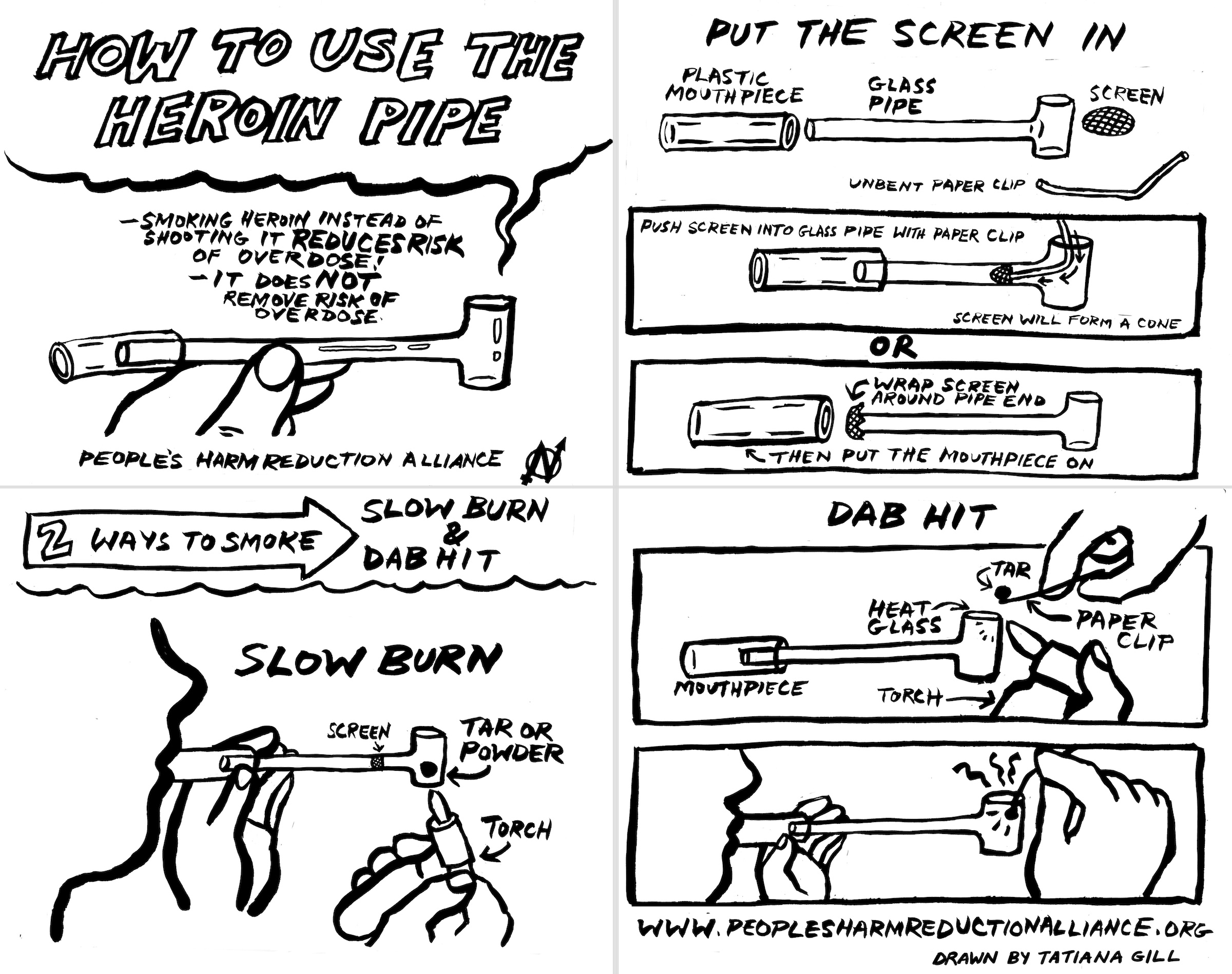

Supplement: Supplementary file 3 — Additional file 3: Educational materials distributed to SSP clients describing how the pipe could be used to smoke heroin. [file 12954_2022_685_MOESM3_ESM.jpg]
