# Supplementary material for: Heroin pipe distribution to reduce high-risk drug consumption behaviors among people who use heroin: a pilot quasi-experimental study
Source: Harm Reduct J. 2022 Sep 22;19:103. doi: 10.1186/s12954-022-00685-7 (PMC9493152; doi:10.1186/s12954-022-00685-7)
Supplement: Supplementary file 4 — Additional file 4: Supplementary tables. [file 12954_2022_685_MOESM4_ESM.docx]

**Supplementary Materials**

**Supplementary Table 1: Number of surveys completed by unique SSP clients**

|  | **No.** | **%** |
| --- | --- | --- |
| **All SSP clients  (n=694)** |  |  |
| Total number of surveys completed |  |  |
| *1* | 554 | 80% |
| *2* | 78 | 11% |
| *3* | 31 | 4% |
| *4* | 12 | 2% |
| *5* | 11 | 2% |
| *6* | 5 | 1% |
| *7* | 3 | 0% |
| Completed one or more survey in both the pre- and post-intervention period |  |  |
| *Yes* | 96 | 14% |
| *No* | 598 | 86% |
| **SSP clients who used heroin at any survey timepoint (n=408)** |  |  |
| Total number of surveys completed |  |  |
| *1* | 323 | 79% |
| *2* | 53 | 13% |
| *3* | 19 | 5% |
| *4* | 8 | 2% |
| *5* | 3 | 1% |
| *6* | 2 | 0% |
| *7* | 0 | 0% |
| Completed one or more survey in both the pre- and post-intervention period |  |  |
| *Yes* | 56 | 14% |
| *No* | 352 | 86% |

**Table 2: Sociodemographic characteristics and drug use behaviors between SSP clients during the pre-intervention and post-intervention periods**

|  | **Pre-intervention (n = 360)** | | **Post-intervention (n = 430)** | |  |
| --- | --- | --- | --- | --- | --- |
|  | No. / Median | % / IQR | No. / Median | % / IQR | p-value |
| **Sociodemographic characteristics** |  |  |  |  |  |
| Age ^Ⴕ^ |  |  |  |  | 0.73 |
| Years | 36 | 29-49 | 38 | 30-49 |  |
| Gender ^Ⴕ^ |  |  |  |  | 0.33 |
| Male | 251 | 71% | 270 | 68% |  |
| Female | 96 | 27% | 117 | 29% |  |
| Trans / Genderqueer / Non-binary | 5 | 1% | 11 | 3% |  |
| MSM status ^Ⴕ^ |  |  |  |  | 0.23 |
| MSM | 24 | 7% | 19 | 5% |  |
| Race ^Ⴕ^ |  |  |  |  | 0.98 |
| White / Caucasian | 188 | 53% | 219 | 55% |  |
| Black / African-American | 47 | 13% | 52 | 13% |  |
| Hispanic / Latino | 17 | 5% | 14 | 4% |  |
| Native American | 21 | 6% | 26 | 7% |  |
| Asian / Pacific Islander | 10 | 3% | 13 | 3% |  |
| Mixed Race | 56 | 16% | 59 | 15% |  |
| Other | 13 | 4% | 15 | 4% |  |
| Monthly income ^Ⴕ^ |  |  |  |  |  |
| Median income | 196 | 0-1000 | 165 | 0-800 | 0.25 |
| No income | 158 | 45% | 189 | 48% | 0.53 |
| Housing status (at latest survey) ^Ⴕ^ |  |  |  |  | 0.169 |
| Housed | 161 | 45% | 170 | 40% |  |
| Unhoused | 199 | 55% | 258 | 60% |  |
| **Drug use behaviors** |  |  |  |  |  |
| Drug consumption |  |  |  |  |  |
| Exclusive heroin use | 23 | 6% | 33 | 8% | 0.48 |
| Concurrent heroin and meth use (i.e. goofball) | 127 | 35% | 137 | 32% | 0.31 |
| Separate heroin and meth use | 164 | 46% | 202 | 47% | 0.69 |
| Concurrent heroin and cocaine use (i.e. speedball) | 22 | 6% | 29 | 7% | 0.72 |
| Separate heroin and cocaine use | 41 | 11% | 53 | 12% | 0.69 |
| Separate heroin, meth, and cocaine use | 36 | 10% | 42 | 10% | 0.91 |
| Exclusive goofball use | 6 | 2% | 5 | 1% | 0.55 |
| Exclusive meth use | 92 | 26% | 104 | 24% | 0.66 |
| Exclusive cocaine use | 16 | 4% | 16 | 4% | 0.61 |
| Separate meth and cocaine use | 53 | 15% | 66 | 15% | 0.81 |
| Drug administration |  |  |  |  |  |
| Lifetime history IDU ^Ⴕ^ | 289 | 81% | 331 | 77% | 0.28 |
| Number of years since first injection ^Ⴕ^ | 4 | 11-19 | 4 | 10-19 | 0.43 |
| Current IV injection (any drug) | 217 | 60% | 244 | 57% | 0.32 |
| Current IM injection (any drug) | 57 | 16% | 58 | 13% | 0.35 |
| Current smoking (any drug) | 282 | 78% | 345 | 80% | 0.51 |
| ^Ⴕ^ There were 46 missing values for income, 42 for age, 41 for sexual activity, 40 for gender and race, and 2 for lifetime IDU history and past 30-day housing. There were also 28 missing values for number of years since first injected; 27 of these were due to missing age and 1 missing response of age first injected. | | | | | |

**Supplementary Table 3: Comparison of SSP clients who used heroin and self-reported heroin pipe distribution did and did not reduce their heroin injection frequency**

|  | **Self-reported reduced injection (n=61)** | | **Self-reported no impact on injection (n = 189)** | |  |
| --- | --- | --- | --- | --- | --- |
|  | No. / Median | % / IQR | No. / Median | % / IQR | p-value |
| **Exposure to intervention in the past 30 days** |  |  |  |  |  |
| Used heroin pipe (≥1 times) ^Ⴕ^ | 37 | 61% | 73 | 39% | 0.003 |
| Used heroin pipe to smoke heroin (≥1 times) ^Ⴕ^ | 37 | 100% | 54 | 74% | <.001 |
| Unable to procure heroin pipe because out of stock (≥1 times) ^Ⴕ^ | 14 | 38% | 24 | 33% | 0.68 |
| **Sociodemographic characteristics** |  |  |  |  |  |
| Age (years) ^Ⴕ^ | 37 | 10 | 36 | 10 | 0.40 |
| Gender ^Ⴕ^ |  |  |  |  | 0.91 |
| Male | 35 | 61% | 102 | 58% |  |
| Female | 21 | 37% | 70 | 40% |  |
| Trans / Genderqueer / Non-binary | 1 | 2% | 5 | 3% |  |
| MSM status ^Ⴕ^ |  |  |  |  | 0.46 |
| MSM | 1 | 2% | 6 | 3% |  |
| Race ^Ⴕ^ |  |  |  |  | 0.93 |
| White / Caucasian | 36 | 63% | 105 | 59% |  |
| Black / African-American | 3 | 5% | 12 | 7% |  |
| Hispanic / Latino | 1 | 2% | 7 | 4% |  |
| Native American | 4 | 7% | 10 | 6% |  |
| Asian / Pacific Islander | 3 | 5% | 7 | 4% |  |
| Mixed Race | 9 | 16% | 27 | 15% |  |
| Other | 1 | 2% | 9 | 5% |  |
| Monthly income ^Ⴕ^ |  |  |  |  |  |
| Median income | 100 | 0-1000 | 200 | 0-1000 | 0.89 |
| No income | 30 | 53% | 97 | 55% | 0.78 |
| **Heroin consumption behaviors in the past week** |  |  |  |  |  |
| Exclusive IV and/or IM heroin use | 17 | 28% | 63 | 33% | 0.43 |
| Both IV and/or IM heroin use and heroin smoking | 36 | 59% | 76 | 40% | 0.01 |
| Exclusive heroin smoking | 8 | 13% | 50 | 26% | 0.032 |
| **High-risk injection behaviors in past 30 days** |  |  |  |  |  |
| Syringe sharing | 6 | 10% | 17 | 9% | 0.84 |
| Syringe reuse | 18 | 30% | 45 | 24% | 0.37 |
| Public injection | 40 | 66% | 98 | 52% | 0.06 |
| **Drug-related health outcomes in past 30 days** |  |  |  |  |  |
| Non-lethal opiate overdose | 3 | 5% | 15 | 8% | 0.43 |
| SSTI (cellulitis and/or abscess) | 21 | 34% | 53 | 28% | 0.34 |
| Hospitalization (all cause) | 8 | 13% | 30 | 16% | 0.60 |
| Endocarditis | 0 | 0% | 2 | 1% | 1 |
| ^Ⴕ^ There were 16 missing values age, gender, sexual activity, race, and monthly income. There were 2 missing values for being unable to procure a pipe and 1 missing value for using a heroin pipe in the past 30 days and using it to smoke heroin. | | | | | |
